# Biologically Inspired Spatial–Temporal Perceiving Strategies for Spiking Neural Network

**DOI:** 10.3390/biomimetics10010048

**Published:** 2025-01-14

**Authors:** Yu Zheng, Jingfeng Xue, Jing Liu, Yanjun Zhang

**Affiliations:** Beijing Institute of Technology, Beijing 100081, China

**Keywords:** brain inspired, spiking neural network, neuron pairs, time slicing, environment perception

## Abstract

A future unmanned system needs the ability to perceive, decide and control in an open dynamic environment. In order to fulfill this requirement, it needs to construct a method with a universal environmental perception ability. Moreover, this perceptual process needs to be interpretable and understandable, so that future interactions between unmanned systems and humans can be unimpeded. However, current mainstream DNN (deep learning neural network)-based AI (artificial intelligence) is a ‘black box’. We cannot interpret or understand how the decision is made by these AIs. An SNN (spiking neural network), which is more similar to a biological brain than a DNN, has the potential to implement interpretable or understandable AI. In this work, we propose a neuron group-based structural learning method for an SNN to better capture the spatial and temporal information from the external environment, and propose a time-slicing scheme to better interpret the spatial and temporal information of responses generated by an SNN. Results show that our method indeed helps to enhance the environment perception ability of the SNN, and possesses a certain degree of robustness, enhancing the potential to build an interpretable or understandable AI in the future.

## 1. Introduction

**Figure 1:**
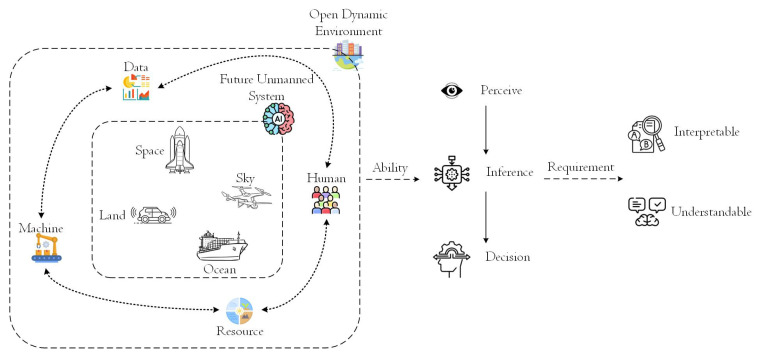
Requirement for future unmanned system.

The real world is a three-dimensional scene covering space, sky, land and ocean, involving elements including people, machines, resources and data. Often, humans will have to make intelligent decisions with incomplete information, uncertain scenarios [1] and uncertain situations. The future autonomous unmanned systems also need to have the ability to perceive, decide and control in an open dynamic environment, especially the ability to make independent decisions in imperfect information scenarios (Figure 1).

With the development of social life moving towards intelligence, the application demand of artificial intelligence (AI) is increasing day by day [2,3,4,5]. Although the current artificial intelligence technology has produced great achievements in image processing [6,7], speech recognition [8], natural language processing and other fields, the ability in reasoning and decision making is still very weak in order to meet the decision-making needs of the future autonomous unmanned system mentioned above.

One of the more important reasons is that current mainstream AI mostly adopts traditional deep neural networks, with a relatively fixed structure, not enough adaptability to different scenes, and not interpretable. Everything can happen in open, dynamic environments. The unknown environmental variables, the unknown variable range of environmental variables and the unknown state of the autonomous unmanned system will bring many unpredictable variables. When there are many unknown variables in the environment, it is necessary to make the autonomous unmanned system have a more robust environment perception ability in advance, so it is necessary to strengthen the training of the environment perception ability. Therefore, it is very important to construct a method with the universal environmental perception ability. Moreover, this perceptual process needs to be interpretable and understandable, so that future interactions between unmanned systems and humans can be unimpeded.

Since the real world contains rich spatial and temporal information, the understanding of both spatial and temporal information is essential for building an understandable or interpretable environmental perception method [9,10,11,12]. However, traditional artificial neural network (ANN)-based AI cannot fulfill the requirement. Many countries have initiated their own brain project to further study the biological brain. It is a common hypothesis that the biological brain, especially the human brain, can be a good teacher to help us to build more generalized and robust AI.

As the core of brain-like AI, the spiking neural network (SNN) has attracted more and more attention nowadays. Spiking signals are the means for an SNN to receive, transfer, hold and express information, which holds both spatial and temporal information essentially. And this is the most distinct and significant feature that makes an SNN superior to traditional ANNs. Through the manipulation of both spatial and temporal information, an SNN has the potential to better understand the characteristics of the real universe, so a better way to implement understandable or interpretable environmental perception.

There have already been many researches about the learning algorithm of the SNN. They can be roughly divided into three categories. The first one is the ANN-to-SNN method, which first trains an ANN, and then converts the ANN to an SNN by substituting neuron models [13,14,15,16,17,18]. The conversion method can achieve almost lossless accuracy compared to its ANN counterpart; however, it lacks the interpretability, which is very important for building trustworthy AI. The second kind of method trains an SNN based on the STDP rule [19,20,21]. However, the STDP-based algorithm is not very efficient for the training of a multi-layer SNN or even a deep SNN. Thus, the third kind of algorithm is proposed, which has explored training SNNs through surrogate gradients to approximate backpropagation, allowing the use of gradient descent. This provides an alternative to STDP-based training [22,23,24,25]. The issue is that they mainly use the spatial information during the training process, which cannot be said to have fully explored the potential of the SNN.

Thus, in this paper, we try to implement a way to better explore the SNN’s potential in understanding both spatial and temporal information, making it more suitable for the implementation of an AI agent for a future understandable or interpretable unmanned system.

The contributions of this work are as follows: (1) it proposes an ROC (Rank Order Coding)-based encoding method, to better capture the spatial and temporal information in the external environment; (2) it proposes a neuron group-based structural learning mechanism to enhance both the spatial–temporal differentiation learning ability and the spatial–temporal information storage ability of an SNN; and (3) it proposes a time slicing-based mechanism to enhance the spatial–temporal differentiation-interpreting ability of an SNN, to help extract the information stored in the SNN, and serve as the basis for building understandable or interpretable AI.

## 2. Related Works

The real world is a world enriched by spatial and temporal information. To better perceive the real world with the neural network means to better recognize important features of information from the world, both spatial and temporal, to have a more efficient way to store information, and to have a more efficient way to extract the information stored in the neural network.

### 2.1. Information Encoding Mechanism of SNN

Encoding method refers to the way that external information been recognized and imported into the neural network. It determines the way neural network sees the external environment, and would influence the features of information that could be recognized from the external environment.

Encoding techniques that have been explored include Undistorted Weighted-Encoding (UWE) [26], temporal coding [27,28,29], direct coding [30], rate codes [31,32], population codes [33,34], sparse codes [35,36] and Gated Attention Coding (GAC) [37]. Each has relative advantages but also limitations compared to Rank Order Coding (ROC) [38], where more important information is transmitted earlier.

Thus, in this paper, the encoding method is designed based on ROC, ensuring the neural network would first receive more important information rather than less important.

### 2.2. Information Storage Mechanism in SNN

The mechanism for how a biological neural network transfers and stores information has been studied for years [39,40,41,42,43]. Xu et al. [44] mimicked the retrospective memory mechanism in neuropsychology and proposed MemoNet, an instance-based approach to predict an agent’s motor intentions by looking for similar scenarios in the training data. Apm et al. [45] presented an adaptive neural network model, demonstrating that the existence of a brief period of relatively high synaptic connectivity is crucial for the development of the system under noisy environments, such that the resulting network can recover the stored memory.

In neuroscience, classical Hopfield networks are standard biological models of long-term memory, relying on Heb plasticity for storage and attractor dynamics for recall. Tyulmankov et al. proposed the implementation of a basic Key-Value Memory Network [46]. It uses a combination of biologically plausible three-factor plasticity rules to store the input. The performance of the network on the self-associative memory task is the same as that in the classical Hopfield network. The network runs in the order of Read, Write key, and Write Value, and consists of three fully linked layers of neurons.

Combining hippocampal architecture and an SNN, Zhang et al. provided a new Biomimetic Spike Temporal Memory (BSTM) model, which includes three layers: the Dentate Gyrus (DG) layer, Cornu Ammonis area 3 (CA3) layer and Cornu Ammonis area 1 (CA1) layer, which are responsible for exploring the encoding, formation and retrieval of episodic memory [47]. All functions of the BSTM model are based on biomimetic spiking neurons, whose biological features include columnar and dendritic structures, emitting and receiving spikes, and delayed transmission.

However, those works almost all adopt the fixed structure, which actually limits the potential of an SNN to represent spatial and temporal information. Thus, in this work, we attempt to enhance the ability of the SNN to store both spatial and temporal information with the help of a neuron group-based structural learning mechanism.

### 2.3. Information Extraction Mechanism of SNN

Cognitive functions are all mediated by networks of many cortical sites whose activity is orchestrated by complex temporal dynamics. To understand cognition, we need to simultaneously identify the brain’s responses in both space and time [48].

Many researches have been targeted at revealing the information extraction mechanism of the human brain, usually studied in neuroimaging, analyzing the cerebral behavior triggered by mental activities. In neurophysiology, Multivoxel Pattern Analysis (MVPA), standing for ‘decoding’, analyzes a joint of voxels simultaneously rather than one voxel per time. MVPA was first introduced to enhance functional MRI (fMRI) data by adding a time dimension to the space data [49]. Later, MVPA was applied in the ElectroEncephaloGram (EEG) and MagnetoEncephaloGraphy (MEG). Commonly used techniques in MVPA are Common Spatial Pattern (CSP) [50], Source Power Comodulation (SPoC) [51] and the Temporal Generalization Method (TGM) [52].

However, when interpreting from an SNN, most SNN studies aggregate neuronal activities into a simplified collective representation, obfuscating temporal dynamics. For example, in typical classification problems, a common practice is to represent each class with a specific output neuron. Existing interpreting schemes include: (a) voting the one that spikes the most [53,54]; (b) voting the one that spikes the earliest [55]; (c) voting the one that has the highest average firing rate [56]; etc. These approaches have obliterated the bionic advantage of the SNN. In recent years, some works have also been proposed to explore how to better interpret the SNN inspired by findings in biological research, such as the work of Wu et al. [30] in which they converted temporal information that an individual neuron conveyed to the spatial correlations of a neuron population, where each class had its voting population. Advanced techniques like multivoxel pattern analysis from neuroimaging could better decode spatial–temporal information from SNNs [57].

In this work, the information extraction method is designed based on our opinion on how spatial and temporal information are represented in the SNN.

## 3. Materials and Methods

### 3.1. Network Structure

In this work, we try to provide a way to better explore the spatial and temporal information storing, representation and extracting potential of an SNN. To reduce the impact of the network itself, we built a simple shallow network. We examined our method on a 2-layer network and a 3-layer network.

**Figure 2:**
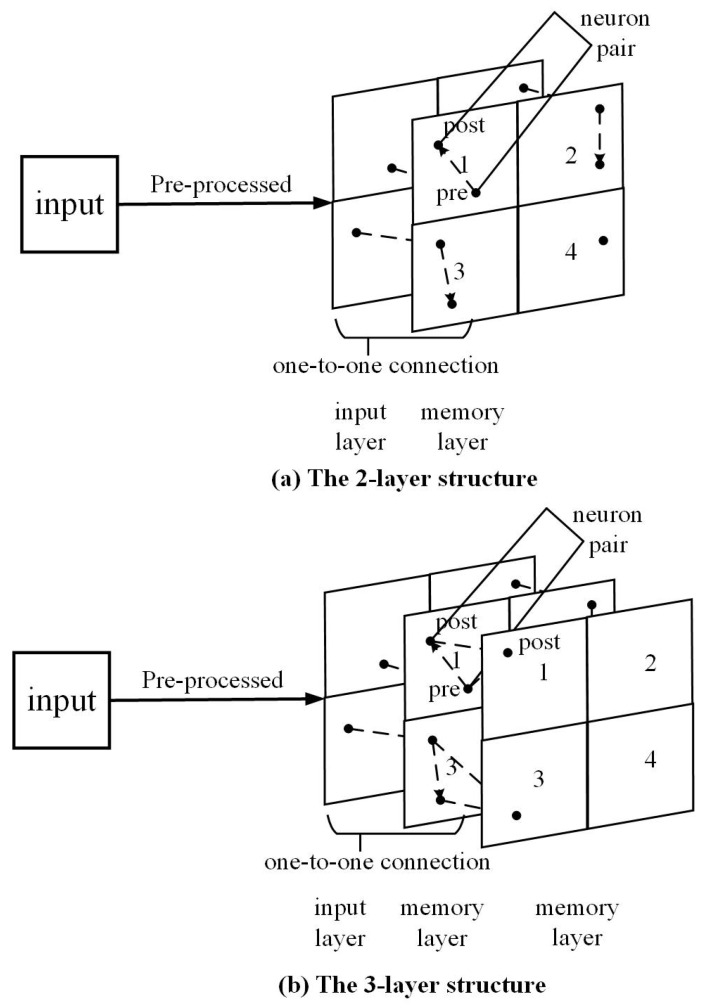
The structure of our 2-layer and 3-layer SNN.

The 2-layer network (Figure 2a) consists of an input layer and a memory layer. Input data would be pre-processed, then fed to the input layer. Connection from the input layer to the memory layer is in a one-to-one manner. The neuron group of a pre- and post-neuron pair forms in the memory layer. Section 3.3.1 discusses this further.

The 3-layer network (Figure 2b) consists of an input layer and 2 memory layers. Input data would be pre-processed, then fed to the input layer. Connection from the input layer to the first memory layer is in a one-to-one manner. The neuron group of a pre- and post-neuron pair and a third post-neuron would form, with the pre- and post-neuron pair in the first memory layer, and the third post-neuron in the second memory layer.

### 3.2. Data Pre-Processing and Encoding

The MNIST dataset is chosen for evaluation. We designed an exponential encoding method complying with Rank Order Coding (ROC) [38]. It converts the value $V_{k}$ of a pixel *k* to a spike arrival time $T_{k}$, given an interval of arrival time from $T_{start}$ to $T_{stop}$,

(1) $$T_{k}=\left(0.5^{V_{k}-1}\right)\times \left(T_{stop}-T_{start}\right)+T_{start}$$

Images were pre-processed before being encoded into spikes. Each 28×28 image was handled with four convolutional filters, and a maximum pooling layer, thus being scaled down to four 12×12 image blocks. The pre-processed image is then directly mapped to the input layer spatially. In this way, the position of a pixel in the image represents the spatial information. And since we transfer the value of a pixel into the spiking time for that pixel, the value of that pixel is now treated as the temporal information. All the layers are composed of 576 neurons.

There are already many different kinds of spiking neuron models, such as Hodgkin–Huxley, SRM, Integrate-and-Fire (IF), Leaky Integrate-and-Fire (LIF), Izhikevich and so on. The LIF neuron model [22,58,59] not only possesses the basic firing and reset effects but also simulates the process of membrane potential leakage when neurons are not stimulated (this process effectively avoids the error of continuous accumulation of membrane potential leading to distortion when neurons are subjected to high-intensity stimulation, compared to the IF model). This ensures the biological similarity of the model without making it too complex to increase the difficulty of simulation. The LIF model is simpler than the Hodgkin–Huxley model and more accurate than the IF model. With its simple computational process and guarantee for key neural activities, LIF is one of the most popular neuron models in the current SNN field. Thus, in this work, we choose to use the LIF model.

### 3.3. Neuron Group Mechanism

The learning process of an SNN is actually a process where the SNN tries to resonate with the external data flows, which represent the real world, by re-constructing its structure topology and re-adjusting the synapses’ weight.

The SNN state $C_{0}$ renews itself iteratively during the constant interaction with the data flow D and gradually forms a relatively stable state $C_{i},\;i>0$. This state is the neural network’s concept of the received data flow.

(2) $$D_{i}\times C_{i}\to C_{i+1}$$

The previous state of an SNN can be referred to as experience or prior knowledge, and it would slightly influence the learning process.

#### 3.3.1. Neuron Pair Formation

The MNIST dataset has 10 categories. As shown in Step 1 of Algorithm 1, we would feed the set of images associating with each digit separately to the SNN. The neuron pair is formed based on Hebbian’s rule [60], which stated that neurons that fire together wire together. In this step, three arguments were required: the time threshold, the distance threshold and the occurrence threshold. If two neurons A and B settle nearby (lower than the distance threshold) and fire within a time interval (lower than the time threshold) frequently (higher than the occurrence threshold), they would form a neuron pair. The thresholds for time, distance and occurrence during neuron pair formation are selected through experimental evaluations.
**Algorithm** **1** Neuron Group Formation.**Step 1:** Neuron pair formation**Input:**        time threshold $T_{thre}$, distance threshold $S_{thre}$, occurrence threshold $C_{thre}$.**Notation:** the first memory layer $M_{1}$, MNIST data *D*, set of neuron pairs *E*. for *digit* in 0–9: Initiate $E_{digit}$; Feed images from $D_{digit}$ to the network; for neuron $N_{i}$ on $M_{1}$:  for neuron $N_{j}$ in the neighborhood $S_{thre}\times S_{thre}$ of $N_{i}$:   if $\mathrm{count}\left(T_{thre}>=\left(T_{N_{j}}-T_{N_{i}}\right)>0\right)>=C_{thre}$:     Add $\left(N_{i},N_{j}\right)$ to $E_{digit}$; Add $E_{digit}$ to *E*;return *E*;**Step 2:** Neuron pair refinement**Input:**        *E*.**Notation:** set of synapse weight of neuron pairs *W*, neuron pair *p*, set of neuron pairs *P*. for digit in 0–9: Initiate $W_{digit}$; Feed images from $D_{digit}$ to the network; Record $W_{digit_{p}}$; Reset synapse weights;Refine *P*;return *P*;**Step 3:** Neuron group formation**Input:**        *P*.**Notation:** the second memory layer $M_{2}$, presynaptic neuron in *p*$N_{pre}$,                   postsynaptic neuron in *p*$N_{post}$. **A.** 3-layer networkfor *p* in *P*: Connect $N_{pre}$ on $M_{1}$ to $N_{post}$ on $M_{2}$; Connect $N_{post}$ on $M_{1}$ to $N_{post}$ on $M_{2}$;Monitor on both $M_{1}$ and $M_{2}$ **B.** 2-layer networkMonitor on $M_{1}$;

#### 3.3.2. Neuron Pair Refinement

The neuron pair needs to be trained before refinement. This is an unsupervised process. Synapse weights between neuron pairs were recorded when each round of a digit was conducted and were reset before the next round.

The refinement of neuron pairs is performed according to several rules: (1) For a neuron pair, if its synapse weight training with samples of a specific digit overwhelmed training with others (higher than a threshold), it would be selected for this digit, otherwise, it would be removed. (2) If several neuron pairs share a same post-neuron, only the neuron pair with the largest weight would be retained.

#### 3.3.3. Neuron Group Formation

The neuron group formation method is different for 2-layer and 3-layer networks. For the 2-layer network, the neuron group is formed simply by the neuron pair after the refinement operation of Step 2 in Algorithm 1. For the 3-layer network, synapses would form from both the pre-neuron and post-neuron in the first memory layer to the corresponding post-neuron in the second memory layer.

### 3.4. Time-Slicing Mechanism

In the process of learning, the human brain will abstract and generalize the characteristics of similar items, extract common features and use them to express these kinds of items. We can name this common feature used to represent the concept of those kinds of items as the ‘expression’ of those kinds of items in the brain. During the training process, we actually want our SNN to capture and abstract the common feature of a category of items, which we call ‘expression’ as the abbreviation of ‘memory expression’, which refers to the common spatial–temporal feature when the SNN receives a category of items as input. When a new sample comes, the criterion to classify a sample is to calculate similarities, or distances, between the sample and the expression of different categories—the category with the minimum distance votes.

The neuron group mechanism helps to enhance the spatial–temporal differentiation learning ability of the SNN, tuning the SNN to better resonate with external data flow input, representing the information differentiation with the structure topology modification and synapses weight adjustment process. However, the mainstream strategy for information extraction from an SNN tends to use aggregated neuronal activities over the simulation into a single indicator that reduces dimensionality, which undoubtedly is not the best way to capture the spatial–temporal differentiation of information stored in the SNN’s structure topology and synapses weight.

We introduce the time-slicing mechanism in this paper to enhance the spatial–temporal differentiation interpreting ability of the SNN. There are spatial and temporal information in the SNN. Time slicing aids in spatial–temporal differentiation, and can better capture the spatial–temporal differentiation of information stored in the SNN’s structure topology and synapses weight, so it helps to better interpret and understand how the SNN represents the information and feature of the environment in itself. Suppose the spatial–temporal differentiation of input data flow has already been captured by the structure topology and synapses weight of an SNN, when new input arrives, the SNN would be evoked to generate spike responses according to the structure topology and synapses weight differentiation of it, which represents the differentiation in pre-learned information. We think this helps to provide a way for us to better understand why the SNN makes such a decision, thus improving the interpretability.

Time slicing aims to disperse spikes on several maps under sliced time intervals. The specific approach is to divide the time window into multiple time slices. Let the time window be *T*, and *S* be the set of all spiking information within *T*. Suppose we adopt an *n*-map time-slicing scheme, then we divide *T* into *n* slices, with each slice having a time interval from $T_{i}$ to $T_{i+1}$. Then, superpose spikes occurring within the sub-interval between $T_{i}$ and $T_{i+1}$ over time, to form a spiking map $M_{i}$ which consists of the spiking set $S_{i}$ within this time slice. A collection of maps retrieved in temporal order helps to interpret the SNN’s responses.

**Figure 3:**
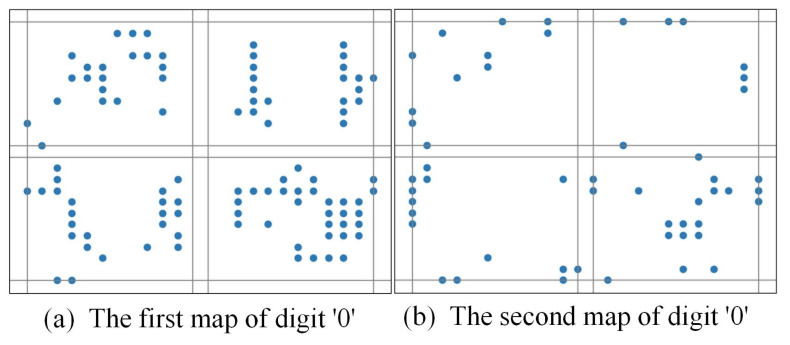
The pattern of an image labeled for digit ‘0’ with a 2-map slicing scheme.

**Figure 4:**
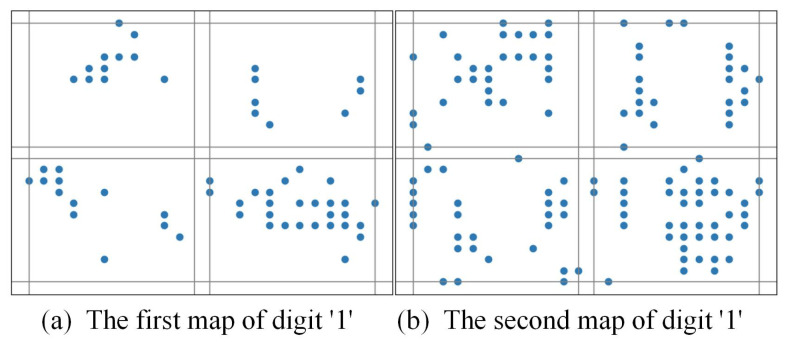
The pattern of an image labeled for digit ‘1’ with a 2-map slicing scheme.

For example, Figure 3 shows that if a 2-map slicing scheme is adopted, the patterns of an image labeled for digit ‘0’ are seen, while Figure 4 shows that if a 2-map slicing scheme is adopted, the patterns of an image labeled for digit ‘1’ are seen. Through the comparison of these two pictures, it can be seen that time slicing could indeed help to better differentiate the difference here.

**Figure 5:**
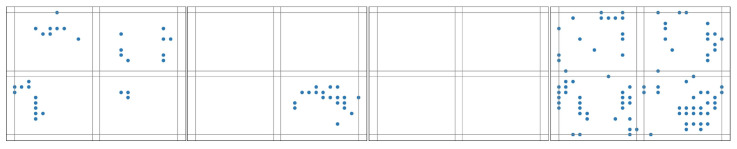
The pattern of an image labeled for digit ‘7’ with a 4-map slicing scheme.

**Figure 6:**
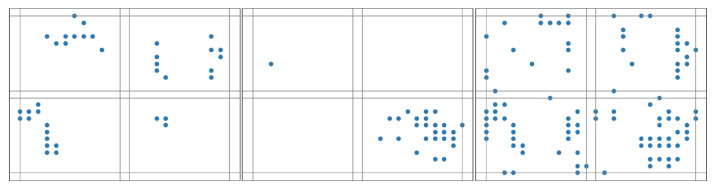
The pattern of an image labeled for digit ‘7’ with a 3-map slicing scheme.

**Figure 7:**
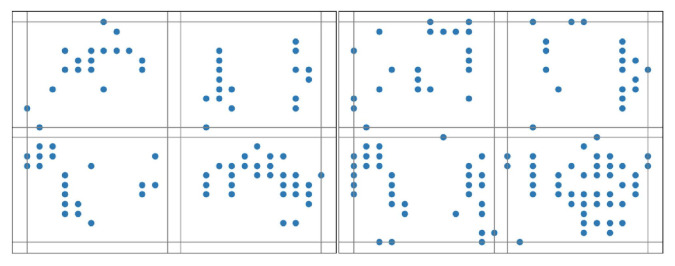
The pattern of an image labeled for digit ‘7’ with a 2-map slicing scheme.

We can also vary the number of time-slicing windows to help capture the spatial–temporal differentiation. Figure 5, Figure 6 and Figure 7 illustrate the results when three different slicing schemes are adopted for pattern extraction of the same image that is labeled for digit ‘7’. Slicing windows are evenly segmented.

Using MNIST as an example, assume we use an *n*-map time-slicing scheme. There are many samples for each digit, and for each sample, *n* spiking maps can be generated. For a specific time interval, the spiking information of all the samples of one digit are aggregated and normalized. Then, for a given firing frequency threshold, select a batch of neurons with more excitation occurrences than the threshold and generate an aggregated spiking map. In fact, this batch of neurons represents the significant features of that time slice for a digit. This process is applied to other time intervals in turn; then, the *n* aggregated spiking map would be generated. In this work, we call the *n* generated aggregated spiking map the ’expression’ of a digit *k*.

Suppose $N_{k}=\left\{M_{k1},M_{k2}\ldots M_{kn}\right\}$ represents the *n*-map collection expression of the digit *k*, and $N^{\prime }=\left\{M_{1}^{\prime },M_{2}^{\prime },\ldots M_{n}^{\prime }\right\}$ is the new sample. Then, we calculate the Jaccard distance $\delta (N_{k},N^{\prime })$ between $N_{k}$ and $N^{\prime }$ as

(3) $$\delta \left(N_{k},N^{\prime }\right)=\sum_{i}^{n}\left(1-\frac{|M_{ki}\cap M_{i}^{\prime }|}{|M_{ki}\cup M_{i}^{\prime }|}\right)$$

The best match satisfies $\min_{k=0,1,\ldots ,9}\delta \left(N_{k},N^{\prime }\right)$. Distances can also be applied in selecting proper slicing schemes.

**Figure 8:**
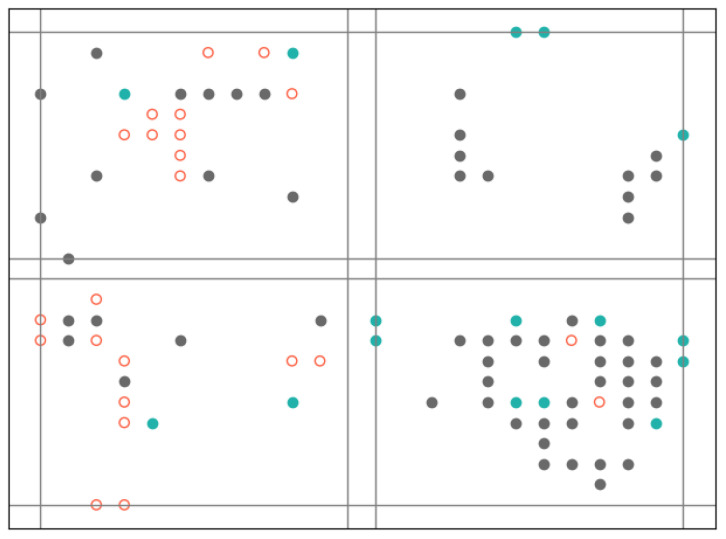
Analogizing a sample as a signal.

Figure 8 is an example of how a sample correlated with an expression. The gray dots are activated neurons that both appear in the expression slice and the sample slice. The red dots are activated neurons in the expression slice but not included in the sample slice. The green dots are activated neurons only in the sample slice. Thus, the expression consists of gray dots and red dots, while the sample consists of the gray dots and green dots.

## 4. Results and Discussion

The experiment aims to (a) evaluate the effectiveness of the time-slicing scheme on our synaptic combination algorithm, (b) evaluate the firing frequency of neuron pairs classified as memory expression in the process of memory formation and (c) evaluate the feasibility to add preset currents to improve the usability of time slices.

### 4.1. Experiment Settings

The experiment is carried out on the NEST simulator [61], tested with the MNIST dataset [62]. Digit expressions were formed after the observation of 10×200 samples.

Spike-Timing-Dependent Plasticity (STDP) synapses are used for training, while static synapses for testing. The learning process was unsupervised, which depended on the intensity of the synaptic connection.

Parameter settings are given in Table 1. As spikes would be delayed, the simulation interval is set 20 ms greater than the encoding interval to cover all spikes within a simulation period.

### 4.2. Evaluating the Time-Slicing Scheme

**Figure 9:**
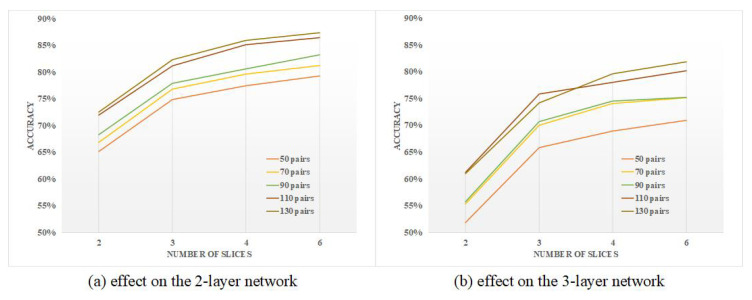
Accuracy improvement for more than two slices.

The time-slicing scheme is examined with a varying number of neuron pairs connected in the input layer. As one simulation duration was 120 ms, the duration is evenly split to 2, 3, 4 and 6 slices, corresponding to 60 ms, 40 ms, 30 ms and 20 ms unit time. Results are given in Figure 9. Accuracy improves as unit time reduces. The effect on the 3-layer network is more obvious than on the 2-layer network, especially for fewer neuron pairs. This can be measured by the dissimilarity (distance) between a sample and the expression. The dissimilarity of patterns is additive, which can be amplified by adding the distance of a single map.

**Figure 10:**
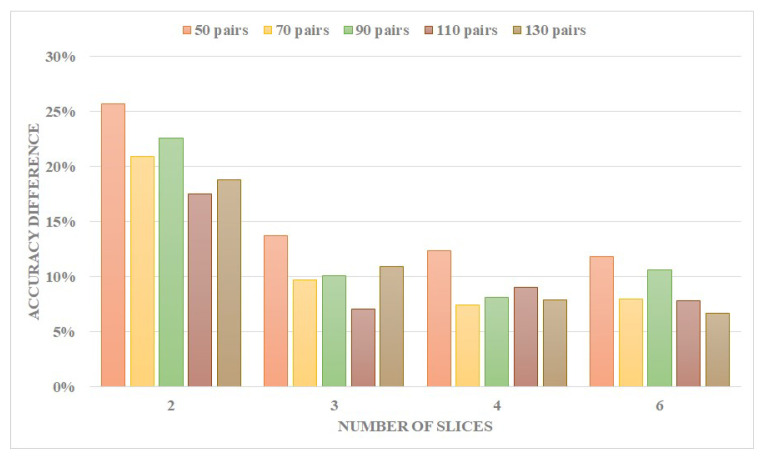
The effect of number of neuron pairs on the accuracy difference of 2-layer network and 3-layer network.

In addition, accuracy is closely attached to the number of neuron pairs. A signal is weakened when transmitting through layers. The effectiveness of the signal reaching the final layer decreases sharply; the amount of this is not enough to represent the input precisely. Accuracy in the 3-layer network is less than the counterpart of the 2-layer network. Generally, when there are more neuron pairs, the 3-layer accuracy approaches closer to the 2-layer accuracy, as demonstrated in Figure 10. Additionally, as unit time per slice increases, accuracy gaps between the two networks become smaller.

### 4.3. Evaluating the Firing Frequency Threshold When Forming Memory Expression

#### 4.3.1. Result for MNIST

**Figure 11:**
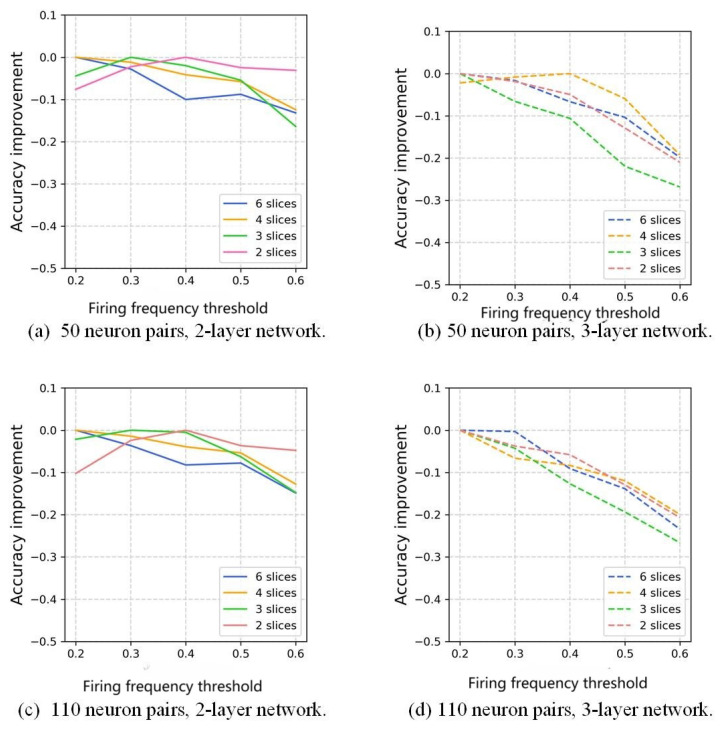
The effect of neuron pairs with different firing frequencies in the process of memory formation on accuracy for MNIST.

According to our time-slicing method, after being aggregated and normalized, only neurons with more excitation occurrences than the firing frequency threshold would be selected to form the memory expression, so the choosing of a firing frequency threshold would have an influence on the result. The change trend of the accuracy for different firing frequency thresholds compared with the highest value is illustrated in Figure 11.

Examples of the number of neuron pairs are 50 and 110. Every poly-line represents an accuracy reduction or negative growth, in comparison to the control groups, i.e., the highest values that equal to 0. For instance, in the six slices scheme in Figure 11b, the accuracy of the frequency threshold set to 0.2 has the highest accuracy, where the group of 0.4 is 10% less. For the highest accuracy, in the 2-layer network, more slices correspond to a lower frequency threshold; fewer slices will shift the threshold higher. Evidently, the high firing frequency raises the characteristics that are weakened by the small number of slicings. However, in the 3-layer network, trends drop sharply as the frequency increases. Again, in this circumstance, involving lower frequently fired neuron pairs enhances the possibility of spiking appearance at the last layer.

Neuron pairs fired by high frequency represent the characteristics of a specific digit; low-frequency neuron pairs are more tolerant to the displacement and deformation of that digit.

This work uses time slices and Jaccard coefficients for information extraction; therefore, the number of neurons excited within the time slice should neither be too many nor too few, meaning the information density should be moderate: too few would lead to sparsity, unclear features and increased randomness, while too many would result in over-compression, reducing differentiation.

**Figure 12:**
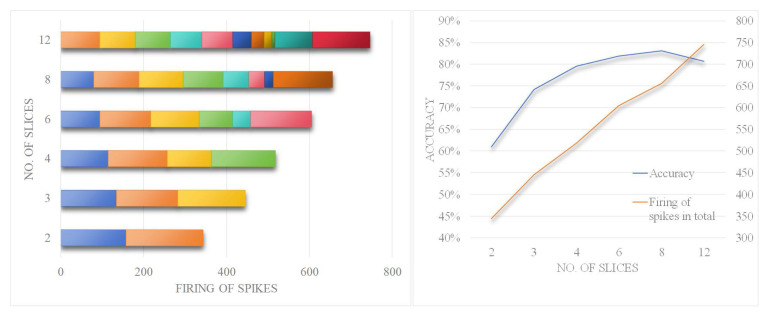
Analysis of excitation amount of different slices.

We conducted further tests on information density. Using a 3-layer network with a firing frequency threshold set to 0.2 and the number of neuron pairs set to 130, we performed detailed analysis of the accuracy changes under different slice counts and presented the average firing rate for 10 digits at each slice count (Figure 12).

Observing the left subgraph, it can be seen that when the number of slices increases, the number of firings of spikes within each slice decreases, whereas when the number of slices decreases, the total number of firings of spikes across all slices decreases. This is because within a time slice, overlapping excitation information is compressed. From the right subgraph, it can be seen that when the number of slices is too small, the compression level is too high, leading to the waste of effective information and low accuracy. As the number of slices increases, the information density reaches an appropriate level. However, when the number of slices further increases to 12, the accuracy decreases, indicating that the amount of information within the slice is insufficient, resulting in increased noise error.

Observing the left subgraph, it can also be found that when the number of slices is 12, there are some time slices with very little excitation, even below 30, and the first slice has no excitation at all. In summary, this experiment further corroborates the aforementioned discussion about the information density.

#### 4.3.2. Result for MNIST-C

**Figure 13:**
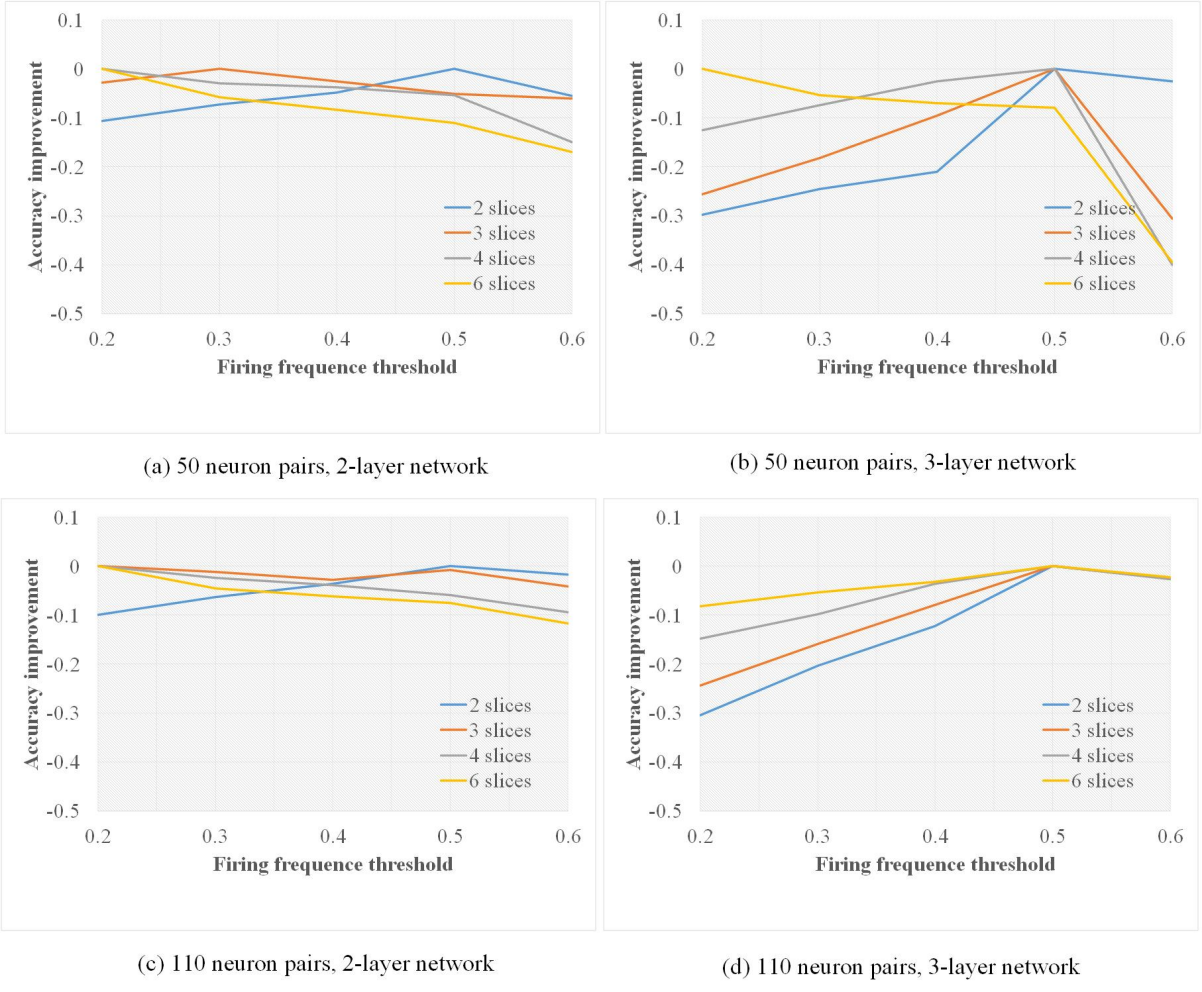
The effect of neuron pairs with different firing frequencies in the process of memory formation on accuracy for MNIST-C.

In order to further evaluate our method, we have conducted experiments on the MNIST-C dataset [63], a benchmark consisting of 15 image corruptions for measuring out-of-distribution robustness. The change trend of the accuracy for different firing frequency thresholds compared with the highest value is illustrated in Figure 13.

It can be seen that there are significant differences in the result of the MNIST-C dataset compared to the MNIST dataset. From Figure 13, it can be observed that for a 2-layer network, the impact of changing the number of slices is not particularly significant. Overall, when the number of slices increases, the smaller the threshold, the better the effect. For the 3-layer network, the impact of changing the threshold is more noticeable. When the firing frequency threshold value is set to 0.5, the performance is generally the best.

### 4.4. Evaluating How Preset Currents Help Improve the Usability of Time-Slicing Scheme

Besides noise, the loss of a sample will greatly affect the identification as well. We added preset currents to make neurons sub-excited. This increases the possibility of signals passing through layers.

We set 100 pA and 300 pA on neurons in the memory layer of the 2-layer network and set the combination of 100 pA and 300 pA in the first and second memory layer of the 3-layer network, respectively, notated as (*a*, *b*) in Table 2. The current firing threshold in our experimental environment is 376 pA. Thus, we set a relatively slight external current at 100 pA and a critical current at 300 pA to pre-activate neurons.

In the 2-layer network, there is a little help with the accuracy if neurons are sub-excited. Approximately 90% of groups with preset currents precede the groups without external currents. The effect of 100 pA is relatively good. Moreover, the higher the frequency of the neuron pairs used when the memory is formed, and the fewer the number of slices, the higher preset currents are needed to make the accuracy higher than no preset currents.

However, the impact is very obvious in the 3-layer network, especially adding to the first memory layer. There are fewer cases where the accuracy of applying external currents merely to the second memory layer is higher than that of no currents added.

Generally, for the 2-layer network, proper preset currents can sometimes slightly enhance image recognition, especially when there are few neuron pairs and more slices. Conversely, preset currents negatively impact accuracy when there are more neuron pairs or few slices. In the 3-layer network, preset currents drastically raise accuracy, where sub-excited neurons help signals propagate further in a multi-layer network.

### 4.5. Evaluating Noise’s Influence

**Figure 14:**
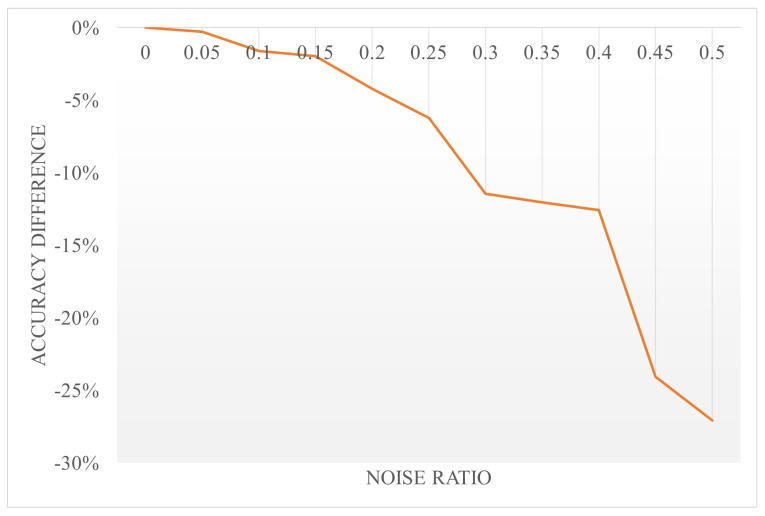
The effectiveness against noise condition.

In order to evaluate our method’s robustness under noisy conditions, we have conducted a set of experiments. We randomly set some of the non-zero pixels in the original image to zero value pixels; that is, the effective information in MNIST images is randomly transformed into noise input, and then we input the processed image into the network to observe the effect. The result is shown in Figure 14. It can be concluded that when the noise ratio is under 20%, the accuracy only decreases slightly. Even when the noise ratio is greater than 20%, the decrease in accuracy is not very significant. It can be seen that our method is quite robust under noisy conditions.

### 4.6. Comparison with Other Methods

The comparison with other methods on MNIST-C is shown in Table 3, demonstrating our method generated a reasonably high result compared to other State-Of-The-Art methods.

## 5. Conclusions

In this paper, we proposed a neuron pair structural learning method to better capture the spatial and temporal information in input data flow to better perceive the features of external environment, and proposed a time-slicing method to better interpret the spatial and temporal information stores in a neural network to help interpret the response of an SNN. This was all for the implementation of the basis for building understandable or interpretable AI.

Our method derives information directly from memory instead of further processing the expression. Therefore, our approach enriches the information compared with single-dimensional methods. Meanwhile, according to our experiment results, the number of neuron pairs in the input layer is critical. A proper amount of pairs express expressions and samples effectively, and sub-excited neurons help signals penetrate multi-layer networks. Furthermore, this method is scalable to complex networks and supervised learning.

There are reported works about Interpretable SNN models, like [70,71,72]. The method proposed in this work could generate new SNN synapses to form a neuron pair, meaning our SNN has a flexible structure; this is the main difference between our method and the Interpretable SNN models presented in [70,71,72]. In our opinion, this topology flexibility is very important for enhancing the ability for an SNN to capture the spatial–temporal feature from the external inputs. Moreover, the time-slicing mechanism is not mentioned in those models.

In our opinion, the proposed neuron pair mechanism can help to better reflect and capture the spatial–temporal feature from the external input. And the proposed time-slicing mechanism could help to better interpret the spatial–temporal differential information stored in the SNN, thus making the SNN more understandable or interpretable.

Our method can be expanded into deeper and more complex networks. The mechanism of the neuronal group can be extended. For deeper networks, chain mechanisms and bypass mechanisms can be introduced to achieve better and faster environmental perception. As for the time-slicing mechanism, the main focus is on exploring the method of selecting slicing time points to maximize the information entropy obtained.

The work in this paper can be the first step towards implementing the ability of universal environment extraction, which can make the autonomous unmanned system have more powerful environment perception ability, so as to solve the problem of the migration of decision-making methods.

## Figures and Tables

**Table 1:** Experimental parameter settings.

Parameter	Value	Unit
Neuron model	iaf_psc_alpha	N/A
Refractory period	1	ms
Encoding time interval	100	ms
Simulation time interval	120	ms

**Table 2:** Accuracy improvement by preset currents.

Neuron Pairs	Slices	2-Layer Network		3-Layer Network	
		Max Group	Acc. Improvement	Max Group	Acc. Improvement
50	6	100 pA	0.45%	(300, 0) pA	43.94%
	4	No preset	N/A	(0, 100) pA	34.41%
	3	No preset	N/A	(100, 0) pA	16.44%
	2	No preset	N/A	(100, 0) pA	1.21%
110	6	100 pA	0.11%	(300, 0) pA	34.08%
	4	No preset	N/A	(100, 0) pA	18.88%
	3	No preset	N/A	(100, 0) pA	6.32%
	2	No preset	N/A	(100, 0) pA	1.02%

**Table 3:** Comparison with other methods.

Method	Accuracy
[64]	80.1%
[65]	81.1%
[66]	77.6%
[67]	82.5%
[68]	87%
[69]	89.3%
Our method	88.4%

## Data Availability

The original contributions presented in the study are included in the article, further inquiries can be directed to the corresponding authors.
